# Glycocalyx and sepsis-induced alterations in vascular permeability

**DOI:** 10.1186/s13054-015-0741-z

**Published:** 2015-01-28

**Authors:** Cosimo Chelazzi, Gianluca Villa, Paola Mancinelli, A Raffaele De Gaudio, Chiara Adembri

**Affiliations:** Department of Health Sciences, University of Florence, Section of Anesthesiology, Intensive Care and Pain Medicine, Viale Pieraccini, 6, 50139 Florence, Italy

## Abstract

Endothelial cells line the inner portion of the heart, blood vessels, and lymphatic vessels; a basal membrane of extracellular matrix lines the extraluminal side of endothelial cells. The apical side of endothelial cells is the site for the glycocalyx, which is a complex network of macromolecules, including cell-bound proteoglycans and sialoproteins. Sepsis-associated alterations of this structure may compromise endothelial permeability with associated interstitial fluid shift and generalized edema. Indeed, in sepsis, the glycocalyx acts as a target for inflammatory mediators and leukocytes, and its ubiquitous nature explains the damage of tissues that occurs distant from the original site of infection. Inflammatory-mediated injury to glycocalyx can be responsible for a number of specific clinical effects of sepsis, including acute kidney injury, respiratory failure, and hepatic dysfunction. Moreover, some markers of glycocalyx degradation, such as circulating levels of syndecan or selectins, may be used as markers of endothelial dysfunction and sepsis severity. Although a great deal of experimental evidence shows that alteration of glycocalyx is widely involved in endothelial damage caused by sepsis, therapeutic strategies aiming at preserving its integrity did not significantly improve the outcome of these patients.

## Introduction

Sepsis is the clinical syndrome of a systemic response to microbial infections. During septic shock, mortality can peak at 56% [1,2]. Sepsis is associated with altered micro-hemodynamics and heterogeneous local perfusion, micro-thrombosis and endothelial dysfunction, alteration of permeability, and interstitial fluid shift [3-6]. The endothelial glycocalyx is a complex macromolecular network involved in many endothelial functions [7]. Sepsis leads to ubiquitous degradation of the glycocalyx, altered endothelial permeability with hypovolemia, hypoalbuminemia, and edema [8,9]. This review focuses on the role of glycocalyx during sepsis.

## Structure of endothelial barrier

**Figure 1:**
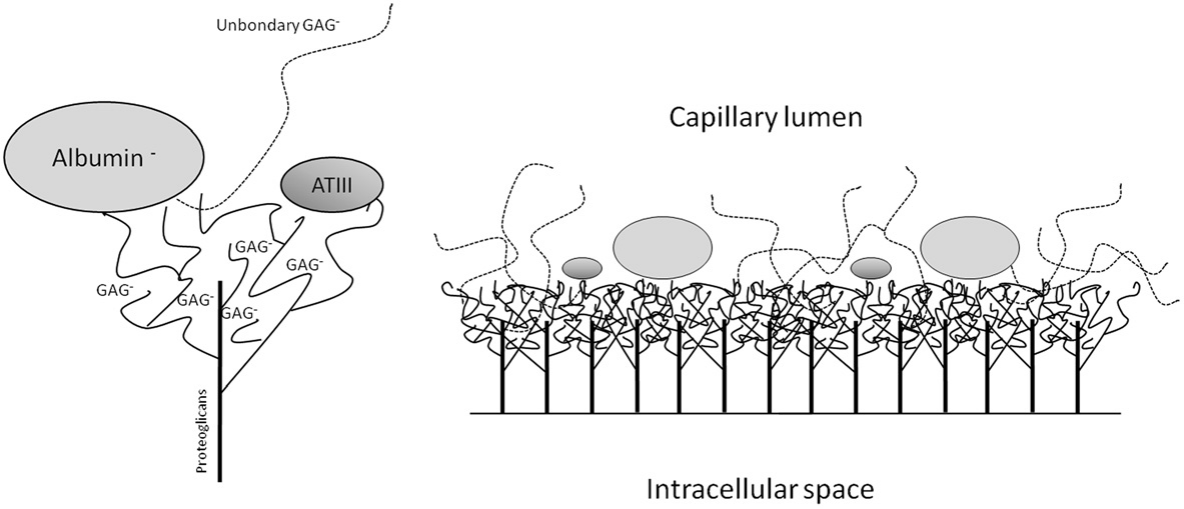
**Glycocalyx structure and the glycocalyx-endothelial barrier.** ATIII, anti thrombin III; GAG, glycosaminoglycan.

Endothelial cells line in a single layer along the inner portion of the heart, blood vessels, and lymphatic vessels. They derive from angioblasts and hemangioblasts and thus are sensitive to the mediators of angiogenesis such as vascular endothelial growth factor (VEGF) [10]. The space between two contiguous endothelial cells is called the endothelial cleft (ETC), which acts as an important site of regulation of endothelial permeability (that is, paracellular permeability) [11]. The apical side of endothelial cells is layered by the glycocalyx, which is 1 to 3 μm in depth (Figure 1). Synthesis of glycocalyx is complex, involving multiple enzymatic pathways; factors regulating its shedding include local pH and mechanical stimuli [12]. Components of glycocalyx include cell-bound proteoglycans, glycosaminoglycan (GAG) side chains, and sialoproteins [4,8,13]. Proteoglycans consist of a core protein to which GAGs are linked. Core proteins include syndecans, glypicans, and perlecans. This complex network envelops endothelial cells on their luminal side and inside the clefts, where it continues into the extracellular matrix of the basal membrane. Soluble components - that is, albumin, unbound hyaluronic acid molecules, thrombomodulin, and various serum proteins (for example, superoxide dismutase, antithrombin III, and cell adhesion molecules) - are bound to the luminal portions of glycocalyx [4].

## Main methods for studying/visualizing glycocalyx

Owing to its fragility and instability, endothelial glycocalyx has been particularly difficult to characterize and understand in its three-dimensional structure [14]. Transmission electron microscopy (TEM), the original method of visualization, has several technical limits and a great deal of effort has been made to reduce them [12,15,16], such as substituting the original ruthenium red staining with Lanthanum or Alcian blue, which allow a better preservation of glycocalyx. The use of non-aqueous vehicles of perfusion is also associated with better preserved samples. Finally, by observing the glycocalyx on rapidly frozen tissues, the alterations due to organic solvents are avoided. A major limit of TEM is that it cannot be used *in vivo*. Another method for direct visualization of glycocalyx is based on fluorescent-labeled ‘lectins’, which are proteins that specifically bind to some components of glycocalyx, or on fluorescent-labeled antibodies for heparan sulfate, syndecans, or hyaluronan. A semi-quantitative measurement of fluorescence can be obtained with a confocal laser scanning microscope in small vessels, whereas in larger vessels two-photon laser scanning microscopy is used because of its increased depth of penetration into tissue [17].

Among indirect methods, intravital microscopy or intravascular volume determinations with permeable/impermeable tracers have been used [18,19].

## Physiologic role of glycocalyx

**Figure 2:**
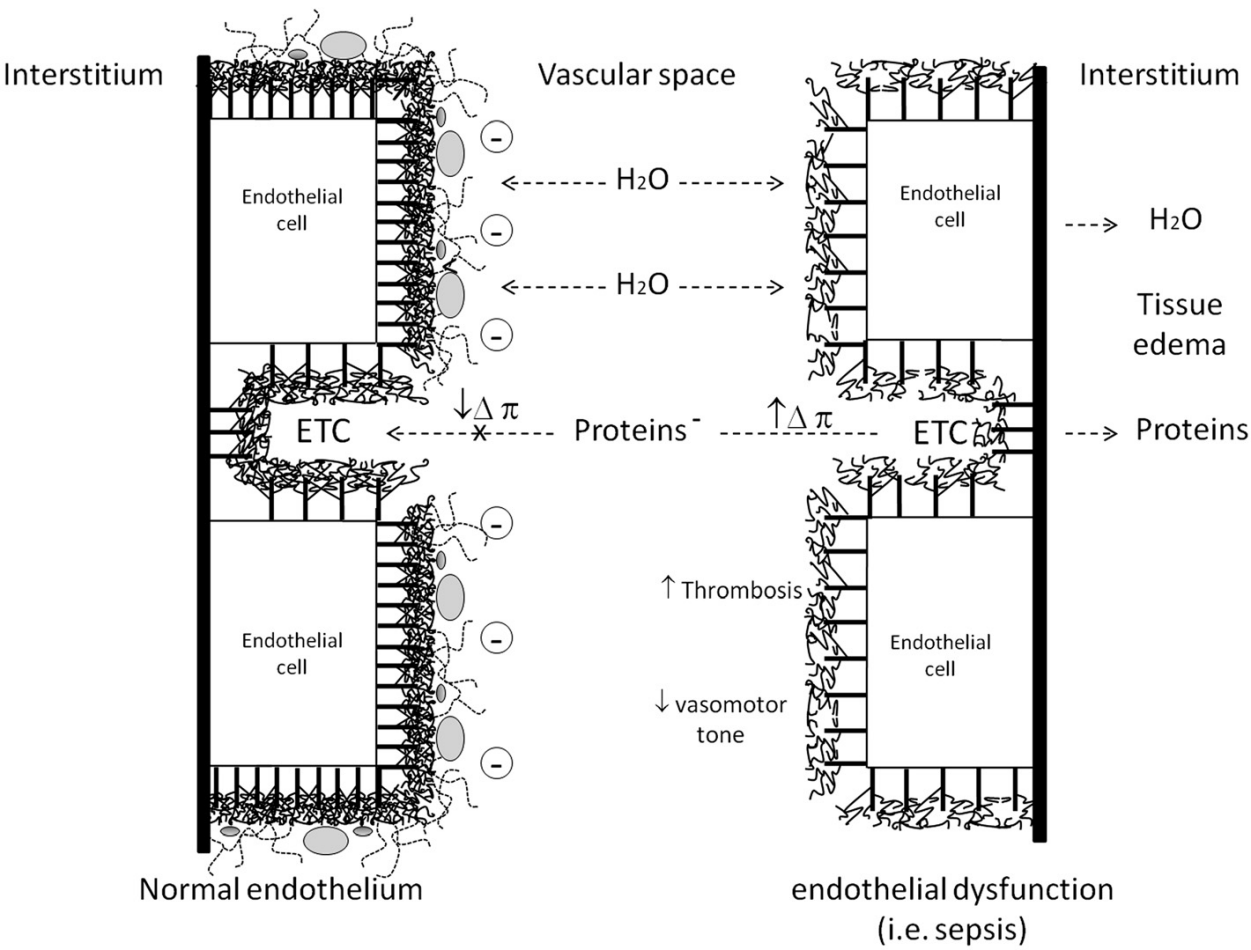
**Glycocalyx characteristics in normal endothelium (left) and during endothelial dysfunction (right).** ETC, endothelial cleft.

The glycocalyx plays a key role in microvascular and endothelial physiology, in particular by regulating microvascular tone and endothelial permeability, maintaining an oncotic gradient across the endothelial barrier, regulating adhesion/migration of leukocytes, and inhibiting intravascular thrombosis [20,21]. The glycocalyx acts as a ‘mechanotransducer’, transmitting shear stress forces to endothelial cells through its intracellular protein domains. Conformational changes in glycocalyx structure lead to release of nitric oxide, thus contributing to regulation of vasomotor tone and peripheral distribution of blood flow/oxygen to tissues [15]. Acting through this mechanism, the glycocalyx contributes to regulation of local blood flow of organs and acts as an effector of metabolic coupling between organ function and local hemodynamics. The glycocalyx is central in maintaining the oncotic gradient across the endothelial barrier. In the so-called ‘paracellular permeability model’, fluids and solutes flow from the intravascular to the interstitial space through the glycocalyx that surrounds the ETCs and reaches the basal membrane. Therefore, the oncotic gradient between intravascular and interstitial spaces is set between the plasma and the glycocalyx rather than being trans-endothelial. In physiologic conditions, the glycocalyx retains a high reflection capability for albumin because of the high density of negative electric charges of its GAG side chains [22]; thus, albumin is not allowed to flow through the clefts (Figure 2). About 40% of total human body albumin is intravascular, whereas only 5% is normally allowed to flow in the interstitial space. Thus, albumin is the main determinant of intravascular colloid-oncotic pressure (πiv). In the paracellular permeability model, the ETCs act as the small pores of the capillaries, through which the transcapillary flow of fluid occurs. In the traditional Starling model, the driving forces for this flow are the intracapillar hydrostatic pressure (Piv) and the interstitial colloid-oncotic pressure (πis). Since the inner portion of the ETCs is covered by the glycocalyx, it was recently proposed that the sub-glycocalyx colloid-oncotic pressure plays a major role as a determinant of transcapillary flow [20]. In normal conditions, this ‘sub-glycocalyx’ space is virtually protein-free, and the only determinant of the transcapillary flow is the Piv. The πiv opposes the flux but does not reverse filtration through the endothelial barrier. In normal conditions, only small amounts of fluid are filtered and most of it returns to the circulation via the lymphatic system. In clinical conditions of increased capillary porosity, like sepsis, albumin and other proteins may reach the extravascular space and, consequently, πis rises and the flow of fluid increases. The use of colloids for volume resuscitation has been associated with increased need for renal replacement therapy, worsening edema, and mortality [23]. This could be due to progressive accumulation of colloidal molecules in the interstitial space through the injured endothelium. In this scenario, functional integrity of the lymphatic system is essential to avoid edema formation [20]. Furthermore, since πiv opposes the fluid transcapillary flux but is not able to reverse it, infusion of albumin or synthetic colloids would not reverse the process of edema formation. This explains why albumin infusion, despite showing a better ‘volemic’ effect, is not able to resolve the interstitial edema in patients with sepsis [20,24]. In hypovolemic states, the fall in Piv leads to a transient back-filtration of fluid from the interstitial space in the intravascular space; as a result, Piv progressively increases until filtration stops. In sepsis, inflammatory injury to the glycocalyx increases porosity of endothelium, and albumin can flow through the ETCs; πiv increases and this further opposes back-filtration of fluid and drives edema formation [20]. Infusion of colloids or crystalloid to restore macro-hemodynamics leads to a progressive increase in Piv that further contributes to trans-capillary fluid escape and interstitial edema. Therefore, the smallest possible volume for plasma volume resuscitation should be used in order to maintain normovolemia and simultaneously reduce the risk of accumulation of interstitial fluid. In sepsis, where porosity increases because of inflammation injury, colloid molecules can flow in the interstitial space, thus increasing πis. These observations may have clinical consequences. Indeed, an interesting recent study on murine models by Bark and colleagues [25] showed that, in conditions of increased transcapillary flow like sepsis, the total volume of colloids necessary to maintain normovolemia can be significantly reduced when administered in slow infusion rather than as a bolus. This effect is probably linked to a sharper increase in Piv that follows quick administration, which in turn leads to greater transcapillary flow.

Molecules of heparan sulfate link antithrombin III, preventing microvascular thrombosis and contributing to maintain microvascular patency. Moreover, the negative charges of GAG side chains inhibit endothelial adhesion of platelets and red cells, further contributing to maintain microvascular anticoagulant properties and rheology. The glycocalyx inhibits leukocyte adhesion to endothelium, thus preventing migration of neutrophils in non-inflamed tissues [7,26,27]. Indeed, integral glycocalyx has a geometry that prevents the interaction between circulating leukocytes and adhesion molecules or selectins. In case of shedding, exposure of these molecules occurs, with subsequent endothelial-cellular interaction [15]. Heparan sulfate binds endothelial superoxide dismutase and xanthine oxidoreductase [28-32]. Thus, intact glycocalyx protects endothelial cells from oxidative stress [20,21].

## Glycocalyx during inflammation

**Figure 3:**
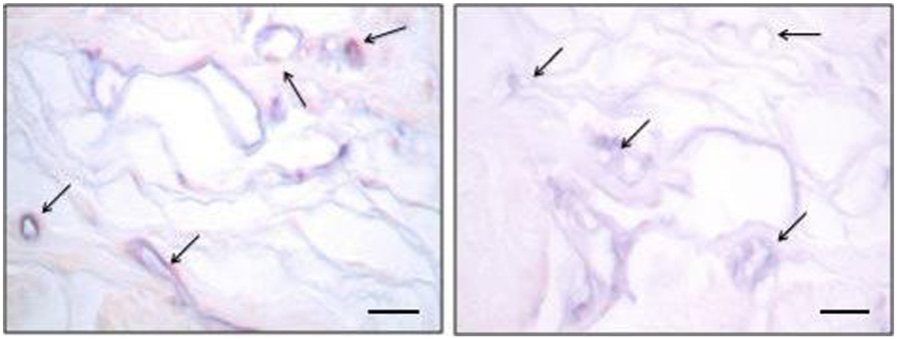
**Modification of glycocalyx components during sepsis.** Representative light microphotograph of MAA (Maackia amurensis agglutinin) lectin histochemistry in mesoceacum of sham-operated (on the left side) and CLP (cecal ligation and puncture)-treated rats (on the right side) at 7 hours after surgery. In blue, MAA reactivity (indicating the presence of sialic acid linked α2,3 to galactose, arrows) was intense in mesoceacal vessels of sham operated rats (left side) and reduced in vessels of CPL rats (right side). Scale bar = 25 μm. Courtesy of Eleonora Sgambati.

Localized or systemic inflammation leads to changes in structure and physiology of glycocalyx, inducing endothelial dysfunction (Figure 3). First and foremost, inflammation injury to glycocalyx is linked to increased paracellular permeability and outflow of albumin/fluid in the interstitial space through the ETCs [20]. Loss of anionic charges, changes in geometry of the clefts, and direct endothelial injury are responsible for this. Other observed changes in glycocalyx function during inflammation include loss of vascular tone with local blood pooling, degradation of heparan sulfate leading to a shift toward a pro-coagulant state with consequent micro-thrombosis, enhanced expression of adhesion molecules with increased leukocyte trafficking, and loss of antioxidative properties with progressive oxidative injury to the endothelium [15]. From an evolutionary and adaptive perspective, this local inflammatory response represents an active and structured response to tissue invasion by micro-organisms or unrecognized cells. In clinical syndromes of systemic inflammation, such as sepsis, major surgery, trauma, ischemia/reperfusion, and prolonged hyperglycemia, diffused and persistent alterations of glycocalyx are linked to widespread endothelial dysfunction, altered permeability, and impaired oxygen and nutrient delivery to cells [8,33,34]. There is experimental evidence that thickness and stiffness of endothelial glycocalyx are reduced by lipopolysaccharide (LPS) or tumor necrosis factor-alpha (TNF-α) exposure [35]. Clinically, these alterations are linked to loss of vascular tone, loss of albumin, hypovolemia, edema formation, and organ dysfunction [7]. Persistence of these alterations is associated with poor clinical outcome even if macrohemodynamic derangements have been corrected (see below). Furthermore, owing to the ubiquitous nature of the glycocalyx, its alterations lead to clinically relevant effects of inflammation also distant from primarily involved organs (that is, ‘organ cross-talk’ as in acute cardiorenal syndromes, where inflammatory injury to kidneys leads to inflammatory damage to endothelium) [36,37].

Exposure to pro-inflammatory mediators such as interleukin 1 (IL-1), IL-2, IL-6, TNF-α, and other molecules released during acute inflammation such as bradykinin, thrombin, VEGF, and histamine results in endothelial activation and massive increase in glycocalyx expression of endothelial leukocyte adhesion molecule 1, intercellular adhesion molecule 1 (ICAM-1), and vascular cell adhesion molecule 1 (VCAM-1). These proteins promote leukocyte rolling, adherence, and migration, which initiate the inflammatory damage to endothelium and tissues [10]. Subsequent damage to endothelium promotes degradation and shedding of glycocalyx with progressive increase in paracellular permeability. During this shedding process, adhesion molecules are released and can be found in circulating blood; thus, in patients with sepsis, circulating levels of VCAM-1 and ICAM-1 parallel those of IL-6 [38]; this was more evident in patients with hyperglycemic sepsis, in which the inflammatory damage to endothelium may be worsened by persistent hyperglycemia [39]. In an experimental model of sepsis, high levels of TNF-α were associated with reduced expression of syndecan 1 and altered composition of hyaluronan and sialic acids [40]. Shedding of the glycocalyx and release of syndecans in the circulation may also retain an adaptive function in limiting the inflammatory damage mediated by TNF-α. Interaction of syndecans with TNF-α leads to a structural rearrangement of endothelial cells and loosening of intercellular junctions; this greatly increases paracellular permeability, further allowing extravasations of fluids, albumin, and solutes [2]. It is known that VEGF induces angiogenesis and markedly increases endothelial permeability [41]. Gram-negative bacterial LPS increases VEGF-mRNA expression in macrophages and induces release of VEGF by leukocytes and platelets [42]. The kidneys are a major source of VEGF production in humans, and it has been speculated that VEGF may play some role in regulating the glomerular permeability to protein. The mechanisms of increased vascular permeability by VEGF may involve stimulation of collagenase production and proteolytic disruption of the endothelial basement membrane [6,43]. Renal release of VEGF during septic acute kidney injury may contribute to distal organ endothelial damage (that is, organ ‘cross-talk’). Therefore, it is important to underline that the urinary microalbuminuria has been demonstrated to be a reliable marker of sepsis-induced alterations of endothelial barrier and changes in systemic paracellular permeability [9,44-48]. Indeed, albuminuria greatly increased in an experimental model of sepsis in association with the aforementioned observed structural changes of glycocalyx [40].

Oxidative stress plays a central role in inflammatory injury to glycocalyx. *In vitro*, exposure of GAGs to reactive oxygen species derived from leukocytes (such as superoxide anion and hydroxyl radicals) causes fragmentation of GAGs with loss of some of its components [49]. Degradation of glycocalyx will expose the endothelial cells to oxidative damage, which has been linked to increased porosity, and interstitial loss of albumin observed in both severe sepsis and chronic conditions like diabetes or hypertension [21,34].

In patients with sepsis, endothelial glycocalyx may be affected by co-existing clinical conditions. Lipoprotein-lipase controls the release of fatty acids to tissues and its primary site of action is the luminal side of the endothelial cell (glycocalyx) [21]; diet-induced hyperlipidemia produces a significant endothelial injury, resulting in transfer of proteins in the sub-endothelial space [50]. Pro-inflammatory cytokines locally released by perivascular adipocytes act through a paracrine mechanism and independently contribute to glycocalyx injury [51]. In contrast, glycocalyx integrity may be protective in atherosclerosis [52], whereas in patients with heterozygous familial hypercholesterolemia, the overall volume of glycocalyx is decreased [53]. Both acute hyperglycemia and chronic hyperglycemia induce shedding of the glycocalyx, contributing to endothelia dysfunction and loss of albumin [54]. It was found that, compared with healthy individuals, patients with diabetes mellitus type 1 or type 2 have a reduced volume of glycocalyx [55]. It is reasonable to argue that acute systemic inflammation observed in sepsis may act in combination with chronic hyperglycemia, insulin resistance, oxidative stress, or activation of the renin-angiotensin system to amplify the already existing injury to glycocalyx. This could make it difficult to compare septic patients with different co-morbidities. The extent of this phenomenon is still unclear, and disease-specific markers of glycocalyx injury have not been described.

## Markers of glycocalyx degradation

Circulating levels of syndecan 1 are related to endothelial damage and glycocalyx degradation and correlate with serum levels of inflammatory cytokines [56]. They are also associated with coagulopathy and increased mortality in trauma patients [57]. Since levels of syndecans may be higher in patients with sepsis than in surgical patients, they are thought to be a biomarker of more extensive damage of the glycocalyx since they are core proteins [38,58]. Endocan is another component of glycocalyx that can be released in response to TNF-α and IL-1 and act as a biomarker in patients with sepsis. Circulating levels of endocan have been shown to correlate with severity of sepsis [4,59], and high serum levels of endocan were associated with the development of acute lung injury after major trauma [60]. As such, endocan is considered a promising biomarker of endothelial dysfuncion in sepsis.

The increase of microalbuminuria is a consequence of increased permeability due to inflammatory injury to glomerular endothelium which is frequently observed in clinical practice and experimental models of septic-related acute kidney injury (see above) [43]. In individual patients, the microalbuminuria-urinary creatinine ratio (MACR) correlates with severity scores such as the Surgical Stress Score [61], the Injury Severity Score [62], Acute Physiological and Chronic Health Evaluation II [63], the Sequential Organ Function Assessment [64], and the Simplified Acute Physiology Score II, and with duration of mechanical ventilation and partial pressure arterial oxygen/fraction of inspired oxygen ratio [65]. In patients with sepsis, MACR levels increase earlier than C-reactive protein and procalcitonin [66]. Indeed, microalbuminuria itself may be considered a marker of sepsis severity [44].

Angiopoietins (Angs) are a novel class of angiogenetic growth factors that have recently been recognized to play a substantial role in the inflammatory process [67]. Ang-1 promotes structural stability of blood vessels, whereas Ang-2 promotes vascular destabilization and permeability [67]. Ang-1 and -2 are reciprocally antagonistic factors involved in important intracellular pathways. In critically ill patients, the release of Ang-2 directly reflects vascular barrier breakdown [68]. It has been demonstrated that Ang-2 is increased in septic shock and that its increase is related to unfavorable outcome [69], although routine measurement of circulating Ang-2 is not clinically feasible.

## Therapeutic strategies

Despite the growing evidence that glycocalyx is extensively involved in sepsis-related altered vascular permeability, strategies aiming at correcting these alterations failed to show clear clinical benefit [3]. Corticosteroids could decrease the inflammatory injury in systemic sepsis and reduce inflammatory damage to endothelium [48]. Indeed, there is some evidence that their use is associated with reduced microalbuminuria; a protective effect on glomerular glycocalyx may be postulated [6,44]. Indeed, low-dose hydrocortisone improved MACR in patients with severe sepsis [44]. Corticosteroids are known to inhibit cytokine synthesis, in particular TNF-α. Since TNF-α leads to rearrangements of endothelial cells (see above), reduced release of TNF-α could preserve the integrity of the endothelium [6]. Despite this experimental evidence, the clinical utility of corticosteroids in sepsis is still debated [70,71]. Anticoagulant molecules such as antithrombin or activated protein C may exert their effects by preserving glycocalyx integrity [72]. However, both molecules failed to improve outcome of patients with septic shock [7]. The use of antioxidant therapies may help to preserve integrity of glycocalyx. One study showed the efficacy of an infusion of N-acetylcysteine in preventing hyperglycemia-induced shedding of glycocalyx [73]. This supports the role of oxidative stress in the impairment of the glycocalyx and in endothelial dysfunction. However, definitive evidence of clinical utility of antioxidant in sepsis is still lacking. Tight glycemic control may reduce glycocalyx shedding in sepsis and preserve endothelial function [21,73]. This potential positive effect of glycemic control has been confirmed also for chronic hyperglycemia [39,74].

## Conclusions

Endothelial dysfunction and glycocalyx degradation are key features of sepsis. In severe sepsis and septic shock, they cause alterations of macro- and micro-circulation, alter organ perfusion, and contribute to cellular hypoxia, which all lead to organ dysfunction. Inflammatory-mediated endothelial injury can be responsible for a number of specific clinical effects of sepsis, including acute kidney injury, respiratory failure, and hepatic dysfunction. Markers of glycocalyx degradation, such as microalbuminuria or circulating levels of endocan or selectins, are promisingly employed as markers of severity of sepsis. Some of the proposed therapies of sepsis, including corticosteroids, may counteract alterations of endothelium and glycocalyx. Their actual clinical efficacy still needs to be proven.

## Abbreviations

Ang: Angiopoietin
ETC: Endothelial cleft
GAG: Glycosaminoglycan
ICAM-1: Intercellular adhesion molecule 1
IL: Interleukin
LPS: Lipopolysaccharide
MACR: Microalbuminuria-urinary creatinine ratio
Piv: Intracapillar hydrostatic pressure
TEM: Transmission electron microscopy
TNF-α: Tumor necrosis factor-alpha
VCAM-1: Vascular cell adhesion molecule 1
VEGF: Vascular endothelial growth factor
πis: interstitial colloid-oncotic pressure
πiv: intravascular colloid-oncotic pressure
